# Community based interventions for strengthening adolescent sexual reproductive health and rights: how can they be integrated and sustained? A realist evaluation protocol from Zambia

**DOI:** 10.1186/s12978-018-0590-8

**Published:** 2018-08-28

**Authors:** Joseph M. Zulu, Isabel Goicolea, John Kinsman, Ingvild Fossgard Sandøy, Astrid Blystad, Chama Mulubwa, Mpundu C. Makasa, Charles Michelo, Patrick Musonda, Anna-Karin Hurtig

**Affiliations:** 1 School of Public Health, University of Zambia, P.O. Box 50110, Lusaka, Zambia; 2 Umeå International School of Public Health (UISPH), Umeå University, 90185 Umeå, SE Sweden; 3 Centre for International Health (CIH), University of Bergen, Bergen, Norway; 4 Center for Intervention Science in Maternal and Child Health (CISMAC), Bergen, Norway

**Keywords:** Realist evaluation, Sexual and reproductive health, Community health systems, Adolescents, Zambia

## Abstract

**Background:**

Research that explores how community-based interventions for strengthening adolescent sexual reproductive health and rights (SRHR) can be integrated and sustained in community health systems, is, to the best of our knowledge, very scarce, if not absent. It is important to document mechanisms that shape integration process in order to improve health systems’ responsiveness towards adolescents’ SRHR. This realist evaluation protocol will contribute to this knowledge in Zambia where there is increased attention towards promoting maternal, neonatal and child health as a means of addressing the current high early pregnancy and marriage rates. The protocol will ascertain: why, how, and under what conditions the integration of SRHR interventions into Zambian community health systems will optimise (or not) acceptability and adoption of SRHR services. This study is embedded within a randomized controlled trial - “Research Initiative to Support the Empowerment of Girls (RISE)”- which aims to reduce adolescent girl pregnancies and marriages through a package of interventions including economic support to families, payment of school fees to keep girls in school, pocket money for girls, as well as youth club and community meetings on reproductive health.

**Methods:**

This is a multiple-case study design. Data will be collected from schools, health facilities and communities through individual and group interviews, photovoice, documentary review, and observations. The study process will involve 1) developing an initial causal theory that proposes an explanation of how the integration of a community-based intervention that aimed to integrate adolescent SRHR into the community health system may lead to adolescent-friendly services; 2) refining the causal theory through case studies; 3) identifying contextual conditions and mechanisms that shape the integration process; and 4) finally proposing a refined causal theory and set of recommendations to guide policy makers, steer further research, and inform teaching programmes.

**Discussion:**

The study will document relevant values as well as less formal and horizontal mechanisms which shape the integration process of SRHR interventions at community level. Knowledge on mechanisms is essential for guiding development of strategies for effectively facilitating the integration process, scaling up processes and sustainability of interventions aimed at reducing SRH problems and health inequalities among adolescents.

## Plain English summary

This protocol aims to explore how community-based interventions for strengthening adolescent sexual reproductive health and rights (SRHR) can be accepted and adopted in community health systems by documenting the conditions that shape acceptability and adoption process of such interventions. Information on such conditions is important for improving health systems’ responsiveness towards adolescents’ SRHR and improving access to reproductive health services among adolescents. Improved access is vital for reducing early pregnancy and marriage rates as well as promoting maternal, neonatal and child health in Zambia. This study is embedded within a randomized controlled trial - “Research Initiative to Support the Empowerment of Girls (RISE)”- which aims to reduce adolescent girl pregnancies and marriages in Zambia through a package of interventions including economic support to families, payment of school fees to keep girls in school, pocket money for girls, as well as  youth club and community meetings on reproductive health.

Data will be collected from schools, health facilities and communities through individual and group interviews, photovoice, documentary review, and observations. These data collection methods will document relevant values, and conditions that shape the acceptability and adoption process of SRHR interventions at community level. Knowledge on such conditions is essential for guiding development of strategies for effectively facilitating the acceptability and adoption process, scaling up processes and sustainability of interventions aimed at reducing SRH problems and health inequalities among adolescents in low and middle-income countries.

## Background

Adolescents (young people aged between 10 and 17 years) have health care needs that are distinct from those of adults, particularly in the area of sexual and reproductive health and rights (SRHR) [1–5]. Neglect of their specific health needs leads to negative outcomes such as unwanted pregnancies, early marriages, sexually transmitted infections, and sexual violence. For example, every year, approximately 7.3 million girls below age 18 give birth in low and middle-income countries (LMICs) while about 10 million girls are married, with 46% of these being in sub-Saharan Africa [6].

Community-based interventions aimed at strengthening SRHR among adolescents can help to reduce such health challenges through making SRHR appropriate and accessible by adolescents [2, 3]. It has long been established that for health services to be beneficial for the young population, they should be adolescent-friendly, that is; accessible, acceptable, equitable, appropriate and effective for different youth subpopulations, as defined by the WHO [4]. For SRHR interventions to be successful, they moreover need to be compatible with the community context and health system structure, that is, they need to be well integrated [5]. A key question for such interventions is therefore: how can they can be integrated and sustained in the local community- and strengthened in health systems? [5].

The community health system is defined as the ‘grey zone consisting of household-level caregivers, volunteers, community leaders and informal health providers, organizational intermediaries such as non-governmental organizations, religious and sporting groups, as well as other government sectors such as housing, education (schools), and social development (11). In most circumstances, community-based health interventions are *“caught between the formal health system and the community and often in a “grey zone” between public, non-governmental and private health systems”* [12]. The formal part includes health system delivery, human resources for health, the supply chain and governance systems. On the community side, key factors to consider include the community’s capacity to engage and participate in the implementation process, commit and sustain health actions and ensure development of effective partnerships between a complex array of actors involved in the intervention. It is thus the combination of the formal health systems and community aspects that make up the community health system [13].

Community-based interventions aimed at providing SRHR information and services can help to reduce SRHR health challenges associated with adolescent pregnancies and marriages. Interventions for reducing adolescent pregnancy, marriage and school dropout have mainly focused on providing SRHR education and economic support, particularly at primary school level [2]. Focusing specifically on SRHR education, positive outcomes were, for example, recorded in an HIV education program in primary schools in Kenya, where the learners were told that the risk of HIV transmission increased with the age of a partner. Compared to the control arm, the intervention sites had 28% lower pregnancy rates [6]. Other interventions have been less successful partly due to poor integration into the community and local health system [5]. For example, the Zimbabwe microcredit program for young women did not effectively meet its overall goals of empowering the SRH capacity of young women. Similarly, an SRH intervention in Malawi did not manage to increase condom use. In Tanzania the impact of interventions on girls and young women’s agency and household gender dynamics was questionable [7].

### Integration of SRHR interventions into the community health system: A missing piece for success

Integration of SRHR services into the community health system is important as it may help make the SRHR services compatible with the local structures and thus appropriate and accessible by adolescents [2, 3].While some countries have taken steps towards integrating SRHR services into the community health system, the pace of the integration progress has been “*generally slow, and little consensus exists about the optimal models of integration or about how best to achieve them, with the consequence that the term means different things to different stakeholders and takes a diversity of forms*” [8]. This problem has been compounded by limited knowledge of the mechanisms for delivering these SRHR interventions, including the integration process and how to sustain changes triggered by the interventions [9]. Thus, there have been increased calls for further research into the mechanisms and contextual factors that support integration and sustainability of such interventions. A growing body of international guidance on the scaling up and sustainable implementation of community-based health interventions, such as interventions to strengthen adolescent SRHR, recognizes the contribution of both the formal health sector and community factors to the success of programmes [9] . However, community health systems remain insufficiently characterized [10, 11]. To enhance the success of such interventions, it is important to promote better integration of community-based health interventions into formal and community aspects of the health systems [13].

### Zambia profile and adolescent sexual and reproductive health

Zambia is a lower middle-income country located in the southern part of Africa. About 60% of the population lives below the internationally recognized poverty line, i.e. on less than $1.90 a day. The country faces numerous health systems challenges. Almost 53% of the total 14 million inhabitants are under the age of 18 years [14]. According to the 2014 Zambia Demographic Health Survey, as many as 31% of those who were aged 20–24 at the time they were interviewed have married before their 18th birthday. In addition, 25% of married girls aged 15–19 have an unmet need for family planning or they are not using any method of contraception. About 30% of girls aged 15 to 19 years have begun child bearing, 8% have experienced sexual violence, and the national HIV prevalence rate among youths aged 15–24 is estimated at 7%. The maternal mortality ratio is still high at 398/100, 000 live births, with about 30% of these deaths being the result of abortions, of which 80% are among adolescents [14].

### The community health system in Zambia

In Zambia, the community health system exists at the level of the health centers and health posts, which are located at the lowest levels of service delivery (primary health care level). These make up the highest proportion of health facilities in Zambia. The health centers and health posts collaborate with community-based health workers such as Community Health Workers (CHWs)/ Community Health Assistants (CHAs) and Neighborhood Health Committees (NHCs) in delivering and monitoring health services (including SRHR services) in the communities.

The community-based health workers are conceived as “members of communities who work either for pay or as volunteers in association with the local health care system, and they usually share ethnicity, language, socio-economic status and life experiences with the community members they serve” [15]. Compared to CHWs, whose training is short and not standardized, CHAs are expected to undergo a year’s standardized training programme [15]. There are about 23,500 CHWs and 1000 CHAs in Zambia. Health posts serve small communities with populations of approximately 500–1000 households in the rural areas. It is this combination of the health centers and health posts and community actors such CHAs/CHWs and structures such as NHCs which make up the community health system. The community component of the Research Initiative to Support the Empowerment of Girls (RISE), which uses schools and communities as the arenas for implementation (described below), will involve CHAs/ CHWs as coordinators in order to initiate integration of the intervention into the community-based health system.

### The RISE intervention in Zambia

The Research Initiative to Support the Empowerment of Girls is a randomized controlled trial funded by the Research Council of Norway, and implemented by the University of Zambia and the University of Bergen from 2015 to 2020 in 157 schools, targeting approximately 4900 girls [16]. The trial enrolled girls who were in grade 7 (average age approximately 14 years) in 2016, and supports them for two years (grades 8 and 9). The trial aims to test interventions for enhancing opportunities for communities to support adolescent girls to continue going to school, and for increasing girls’ possibilities to postpone pregnancy and marriage. The RISE intervention has three arms: 1) the control arm which provides limited school material support (books and pens); 2) the economic arm which supports packages paying school fees, limited monthly financial support to girls, and annual financial support to families; 3) the community component (63 schools), where this project proposal is embedded. The community component includes community and parent meetings promoting supportive social norms around postponement of early marriage and early childbearing as well as promoting education for girls, and establishment of new clubs in order to increase knowledge of SRH including modern contraceptives, and change behavioral and control beliefs relating to contraceptive use among in- and out-of school adolescent girls and boys. The SRH education is delivered at youth clubs twice per month in the school. The community meetings are held twice per term in each school. The community component of RISE aims to decrease unwanted adolescent pregnancies and improve girls’ SRHR through making community health systems more “responsive” towards adolescents’ SRHR needs.

In this paper, we present the protocol of an evaluation to explore the mechanisms of integration which trigger (or not) the strengthening of SRHR for adolescents at community level. Integration is important as it has the potential of facilitating compatibility of SRHR interventions with the local context, and in particular the SHRH needs of the adolescents thus promoting uptake among the adolescents. Such information is essential in understanding some of the differences in achievements in the different contexts/ clusters in the community component as well as serving the implementation of community-based SRHR interventions beyond the RISE intervention.

## Realist evaluation

The methodological approach we have chosen for the project is realist evaluation, which we suggest is the most appropriate for evaluating innovations in community health systems whose implementation involves multiple actors and organizations operating in distinct administrative environments and across multiple layers of hierarchy [17]. Realist evaluation is a type of theory-driven evaluation that aims to ascertain why, how, and under which circumstances programs succeed or fail. It is based on the work of Pawson and Tilley, and it focuses on how the mechanisms of change are triggered by the intervention and contextual factors that lead to observed outcomes [18]. Realist evaluation seeks to provide results that can be acted upon by decision makers.

Realist evaluation begins with the formulation of the theory behind the development of an intervention, known as the *programme theory*. The programme theory is understood as an everyday, prosaic theory that explains how social problems are generated and how interventions can help to solve them. The programme theory is best considered as a hypothesis that can be tested, and that forms the basis for empirical testing in case studies [19]. Usually, programme theories are not explicitly stated when interventions are developed, and consequently the research team have to formulate the programme theory based on previous research and/or on knowledge and experiences of stakeholders involved in the intervention design.

The programme theory is afterwards tested through observation of real cases where the intervention has been implemented. The programme theory connects context (C), mechanisms (M), and outcomes (O) – creating potential ‘CMO configurations’. The application of CMO configurations should result in a set of ‘context-mechanism-outcome’ (CMO) statements: *“In this context, that particular mechanism fired for these actors, generating those outcomes. In that context, this other mechanism fired, generating these different outcomes”* [20]*.* A critical element in realist evaluation is that of *mechanisms*. Mechanisms mediate between the concrete components of the interventions and the outcomes. According to Pawson and Tilley, a mechanism is *“not a variable but an account of the behaviour and interrelationships of the processes that are responsible for the change*” [18]. Elucidating mechanisms has been shown to be a useful way to bridge the gap between theory building and practical recommendations [21]; if we are able to identify the mechanisms that lead to positive change, they can guide scaling-up processes.

Data collected serve to refine the preliminary programme theory and specify a middle-range theory. A middle- range theory *“lies between the minor but necessary working hypotheses … and the all-inclusive systematic efforts to develop a unified theory that will explain all the observed uniformities”* [22]. It provides plausible explanations of why, how, and under what circumstances the intervention triggered particular mechanisms that led to certain outcomes. A theory-driven evaluation is methods-neutral (i.e. it does not impose the use of particular methods) but usually combines qualitative and quantitative methods for collecting data on context, mechanisms and outcomes.

## Conceptual framework

Fostering the integration of an intervention into the community health systems is “both relational and complex” [23] due to a plural set of providers, diverse norms, values as well as less formal and horizontal mechanisms which shape coordination, accountability, health practice and health seeking behaviour at community level [24]. Atun et al., provide a systematic conceptual framework for researching or analyzing the integration of interventions into health systems which will be relevant to our project [5]. According to this framework, examining the integration process requires examining the *nature of the problem* being addressed (e.g. pregnancies and early marriages), the *intervention* (i.e. the RISE community component package)*,* the *adoption system* (e.g. community, households, schools, health facilities), the *health system characteristics* (i.e. Community-based Health Workers, SRHR services*),* and the *broader context* (socio-cultural factors, programme implementers, regulations).

## Method and steps

Drawing on the methods proposed by van Belle et al., we will follow a step-wise approach to the realist evaluation [25]. Mechanisms that shape the integration process of SRHR into community health systems will be developed through the collection and triangulation of data from different sources [26]. Below, the step-wise approach to the realist evaluation is explained in detail.

- *Step 1: Situating the intervention in the context*

The first step adopts a policy analysis approach in which existing relevant policies on SRHR in Zambia and related literature and documentation will be collected and reviewed. Communication with stakeholders will also take place to discuss their understanding of the intervention and jointly agree on the scope of the evaluation.

- *Step 2: Eliciting the preliminary programme theory*

In this step we will develop the programme theory (or hypothesis) to be tested. Based on the discussions and reviews in step 1, we will compile an initial programme theory of integration of the community-based intervention for strengthening adolescent SRHR into the community-based health systems. In line with the realist approach as proposed by van Belle el., this will have two components: 1) an “action model”, outlining the steps and pathways of intervention following a logic model format; 2) a “change model” outlining the developers’ assumptions about how the intervention will work e.g. through development of new systems, collaborative networks etc. This will form the basis for the development of a data collection plan, and data collection strategies. The initial theory will guide the design on empirical case studies.

- *Step 3 Testing the programme theory*

The third step consists of empirically testing the programme theory. Data collected in this step will serve to identify Context-Mechanism-Outcome patterns that provide an explanation for the observed outcomes. For the analysis the “retroduction” approach will be applied, whereby the observed outcomes are explained by looking into the mechanisms and context elements [27]. This step serves to indicate whether the initial programme theory stands as relevant in the light of the empirical findings. We will test the initial programme theory through an in-depth study of the community component by conducting a multiple case study. We aim to select four cases. We shall define cases as the catchment areas of the health posts/ centres and corresponding schools (which can be defined as a unit of the community health system). The cases will be selected on a theoretical replication argument, meaning that their inclusion will be based on their potential to provide contrasting contexts and outcomes. Preliminary strategy for data collection in the cases is presented in Table 1.

- *Step 4: Specification of middle range theory: How, why, for whom and under what circumstances does the innovation work?*

**Table 1:** Data collection strategies

Dimension	Domain	Context	Data collection	Strategy
Mechanisms: Integration of the RISE community component into the community-based health system	Capacity building, motivation, Collaborative action, trust, team work, social networks, social capital	-Stakeholders -Norms, beliefs and values on pregnancy, early marriage and education	- Key informant interviews with CHAs/CHWs, NHC members, teachers, traditional leaders, health workers, peer educators - Photo voice with girls and boys - Community focus group discussions with parents, girls and boys - In-depth interviews with girls, parents	Qualitative semi-structured interviews and checklists
Outcome: Acceptability and adoption (includes accessibility) of SRH services provided by the CHWs/ CHAs at the health post/centre	- Adolescent friendly SRH services -Acceptability of SRH services in the community	- Stakeholders - Norms, beliefs and values on pregnancy, early marriage, family planning and education	- CHW/ CHA records on use of SRH services among youth - Health post records on use of SRH services among peer educators - Key informant interviews with CHAs/CHWs, NCHs, teachers, traditional leaders, health workers, youth - Photo voice with girls and boys - Community focus group discussions with parents, girls and boys - In-depth interviews with girls, parents - Quantitative information on health care seeking from the 6-monthly interviews with the girls	Quantitative and qualitative interviews

Step 4 uses the findings from previous steps to enhance the understanding of how, why, for whom and under what circumstances the integration of sexual and reproductive health and rights (SRHR) interventions into the Zambian community health systems will optimize (or not) acceptability and adoption of SRH services. These are essential questions when moving into the scale-up of interventions. This step will begin concurrently with Step 3 in cycles of reflection. It is anticipated that findings might also become part of action learning processes around specific components (e.g. capacity building of CHWs, team work, work motivation, personal values). The phase will end after data collection is completed and the case studies have been analyzed, and will result in a refined action model of strengthening integration of adolescent SRHR into the community health systems that includes an account of “what worked for whom in the context of the communities in Zambia”.

## Ethical considerations

Ethical approval to conduct the study has been granted by the Excellence in Research Ethics and Science (ERES) in Zambia (Approval number 2018-Jan-007).

 Approval has further been provided by the National Health Research Authority. Voluntary written informed consent, assent and parental or guardian consent will be obtained from all the research participants. During report writing and data analysis, any information that could identify the respondents will not be used.

## Discussion

This paper describes a protocol that uses a multiple case-study design to understand how, why and under which conditions the integration of sexual and reproductive health and rights (SRHR) interventions into community health systems will lead to (or not) acceptability and adoption of SRH services in Zambia. There has been increased use of realist evaluation in health systems research in the recent past [27–33], as it is important in health systems research to assess real circumstances where contextual factors play an important role [27]. Realist evaluation provides such a possibility of a contextual analysis as the approach explores such factors in interaction with the intervention, the outcomes, and the mechanisms.

Understanding the mechanisms that shape the integration of interventions aimed at reducing pregnancies and marriages among girls into community health systems is essential for guiding implementers, policy makers and NGOs in effectively facilitating the integration process and sustainability of interventions into health systems. Improved integration also has the potential for improving SRH outcomes and reducing health inequalities among marginalized groups, and subsequently contributing towards improved socio-economic development of the groups.

However, there are challenges associated with the use of the realist evaluation approach [33–35]. One such issue relates to measuring the effect of the intervention on change in community culture regarding SRHR such as community norms on contraception use and cultural factors that trigger pregnancy and the effects on adolescents. Other challenges include community’s role in service delivery which in our case includes the nature and pattern through which SRH services provided by the community-based health workers have changed [28, 34]. To address such challenges, we plan to use multiple data collection tools. On culture and values, we plan to collect qualitative data through photo voice, in-depth interviews, and focus group discussions. These methods can allow participants the opportunity to express their own views about a topic in terms that they choose and with reference to issues that they consider to be meaningful. However, this does not imply that the methods are inherently better than survey data, as both can play an important role [34].

Regarding service delivery, we plan to collect data using key informant interviews with health providers, as well as in-depth interviews and review of records at the health facility regarding access to SRH services by adolescents. Documentary review may be challenging as some reports may be incomplete, but we expect that they will nonetheless be broadly indicative of the situation. The resulting information gaps will be filled through quantitative and qualitative surveys which will assess the adolescents’ perspectives of the extent to which the current community health system is responsive, and their definition of an ideal or expected responsive adolescent community health system.

Documenting integration into community health systems is difficult as the process is influenced by the “plural set of providers, diverse norms and values, as well as less formal and horizontal mechanisms which shape coordination and accountability at community level” [24, 36], as well as multiple community actors [10, 11, 37]. Though documenting such multiple and complex actors is challanging, other studies have done so using realist evaluation [10, 34, 38]. The realist evaluation is well suited for examining multi-faceted interventions with the diverse actors and components of a program such as this one being evaluated in a contextualized way [28].

## Abbreviations

CHA: Community health assistant
CHW: Community health worker
CMO: Context-mechanism-outcome’
ERES: Excellence in Research Ethics and Science
LMICs: Low and middle income countries
RISE: Research Initiative to Support the Empowerment of Girls
SRHR: Sexual reproductive health and rights
